# The Integrated Transcriptome Bioinformatics Analysis Identifies Key Genes and Cellular Components for Spinal Cord Injury-Related Neuropathic Pain

**DOI:** 10.3389/fbioe.2020.00101

**Published:** 2020-02-19

**Authors:** Runzhi Huang, Tong Meng, Rui Zhu, Lijuan Zhao, Dianwen Song, Huabin Yin, Zongqiang Huang, Liming Cheng, Jie Zhang

**Affiliations:** ^1^Division of Spine, Department of Orthopedics, Tongji Hospital Affiliated to Tongji University School of Medicine, Shanghai, China; ^2^Key Laboratory of Spine and Spinal Cord Injury Repair and Regeneration of Ministry of Education, Orthopaedic Department of Tongji Hospital, School of Medicine, Tongji University, Shanghai, China; ^3^Department of Orthopedics, Shanghai General Hospital, School of Medicine, Shanghai Jiao Tong University, Shanghai, China; ^4^Department of Orthopedics, The First Affiliated Hospital of Zhengzhou University, Zhengzhou, China; ^5^Department of Prevention, Tongji University School of Medicine, Tongji University, Shanghai, China

**Keywords:** spinal cord injury, neuropathic pain, peripheral blood, single-cell sequencing, cellular communication

## Abstract

**Background:**

Spinal cord injury (SCI) is one of the most devastating diseases with a high incidence rate around the world. SCI-related neuropathic pain (NeP) is a common complication, whereas its pathomechanism is still unclear. The purpose of this study is to identify key genes and cellular components for SCI-related NeP by an integrated transcriptome bioinformatics analysis.

**Methods:**

The gene expression profile of 25 peripheral blood samples from chronic phase SCI patients (E-GEOD-69901) and 337 normal peripheral blood samples were downloaded from ArrayExpress and Genotype-Tissue Expression Portal (GTEx), respectively. A total of 3,368 normal peripheral blood mononuclear cells (PBMC) were download from Sequence Read Archive (SRA713577). Non-parametric tests were used to evaluate the association between all of differential expression genes (DEGs) and SCI-related NeP. CellPhoneDB algorithm was performed to identify the ligand–receptor interactions and their cellular localization among single PBMCs. Transcription factor (TF) enrichment analysis and Gene Set Variation Analysis (GSVA) were used to identify the potential upstream regulatory TFs and downstream signaling pathways, respectively. Co-expression analysis among significantly enriched TFs, key cellular communication genes and differentially expressed signaling pathways were performed to identify key genes and cellular components for SCI-related NeP.

**Results:**

A total of 2,314 genes were identified as DEGs between the experimental and the control group. Five proteins (ADRB2, LGALS9, PECAM1, HAVCR2, LRP1) were identified in the overlap of proteins in the significant ligand-receptor interactions of PBMCs and protein-protein interaction (PPI) network based on the DEGs. Only HAVCR2 was significantly associated with NeP (*P* = 0.005). Besides, the co-expression analysis revealed that TF YY1 had significantly co-expression pattern with cellular communication receptor HAVCR2 (*R* = −0.54, *P* < 0.001) in NK cells while HAVCR2 was also co-expressed with mTOR signaling pathway (*R* = 0.57, *P* < 0.001). The results of RT-qPCR and external dataset validation supported the signaling axis with the most significant co-expression patterns.

**Conclusion:**

In peripheral blood of chronic SCI, HAVCR2 might act as a key receptor on the surface of NK cells and interact with ligand LGALS9 secreted by CD14^+^ monocytes, inhibiting NK cells through mTOR signaling pathway and ultimately predicting the occurrence of SCI-related NeP. This hypothetical signaling axis may provide prognostic biomarkers and therapeutic targets for SCI-related NeP.

## Introduction

Spinal cord injury (SCI) refers to the damage to the spinal cord due to trauma, disease or degeneration (Cheshire et al., 1996; Brienza et al., 2018). According to the National Spinal Cord Injury Statistical Center, there are about 12,000 new cases of SCI each year in the United States (Natinal Spinal Cord Injury Statistical Center [NSCISC], 2014). The global SCI incidence is 40 to 80 new cases per million population per year (New et al., 2014). It not only induces locomotor deficits or even complete paralysis physically, but also generates despairing psychological stress (Budh and Osteraker, 2007). Therapeutically, there are no effective treatment strategies for SCI-induced neurological deficits, leading to a high disability rate and adding heavy burdens to the individual family and the whole society. In order to bring tangible benefits to patients with SCI, there is a pressing need to explore the pathologic mechanisms which may provide candidate targets for treatment (Azarhomayoun et al., 2018; Musubire et al., 2019).

Generally, SCI is categorized into three phases: the acute phase (0–15 days), the sub-acute phase (3–5 months) and the chronic phase (6–12 months) (Pouw et al., 2011). Although the functional status of the chronic phase may be considered clinically similar, regardless of the level of injury, new types of pathologies at both micro and macro level occurs involving a variety of aberrant molecules and cellular components, especially immune cells (Metz et al., 2000; Chen et al., 2013; Van Niekerk et al., 2016). The most common pathological features during the chronic phase are the formation of the glial scar resulting from persistent glial activation and neuronal hyperactivity associated with reactive astrocytes, microglias, and infiltrating macrophages (Takeura et al., 2019). In addition, all of these pathological features are related to neuropathic pain (NeP). Thus, we suppose that identifying the mechanism of NeP and predicting its occurrence.

Neuropathic pain is reported to occur in 40–50% of SCI patients and typically develops within the first year following SCI as the chronic presentation (Siddall et al., 2003; Werhagen et al., 2004). Currently, its treatment is difficult and the efficacy of the recommended treatment options are modest (Finnerup et al., 2015). Although the pathological mechanism of NeP is still unclear, it may be associated with the dynamic process of nerve regeneration and immune response (Yune et al., 2007; Lee et al., 2008; Pinzon et al., 2008; Takeura et al., 2019). In addition, no factor has been identified to predict the occurrence of SCI-related NeP. With regard to human specimens, peripheral blood from patients with SCI is the more accessible and minimally invasive than injured spinal cord in clinical practice.

Thus, an integrated transcriptome bioinformatics analysis based on bulk RNA sequence and single-cell RNA sequence was performed to identify differentially expressed genes and cellular communications, key ligand-receptor interactions and their cellular localization in peripheral blood of patients with SCI. In addition, potential upstream transcription factors (TFs) and downstream signaling pathways of key cellular communication genes were also identified to draw a signaling axis, which might provide candidate predictors and therapeutic targets for SCI and SCI-related NeP.

## Materials and Methods

### Data Collection

This study was approved by the Ethics Committee of Tongji Hospital affiliated to Tongji University School of Medicine. The gene expression profile of 25 peripheral blood samples from chronic phase SCI patients (E-GEOD-69901) (Platform: Affy Primeview Gene Expression Array) were downloaded from ArrayExpress^1^ as the experimental group. Because we wanted to identity DEGs between peripheral blood of normal people and patients with SCI, but E-GEOD-69901 did not have the data of normal people. Therefore, we did not use the control set that was published with the E-GEOD-69901. The control group including 337 normal peripheral blood samples was downloaded from the Genotype-Tissue Expression Portal^2^ (GTEx) (Consortium, 2015). A total of 3,368 normal peripheral blood mononuclear cells (PBMC) were download from Sequence Read Archive^3^ (SRA713577) (Freytag et al., 2018). The single cell set was one 10X genomics object of 3,368 cells from the same one person. In order to ensure the repeatability of the experiment, we used the RData file including matrixes of Reads Per Kilobase per Million mapped reads (RPKM) and raw counts from the PanglaoDB (Franzen et al., 2019). Besides, we have carried out an external validation. Two Affy Primeview dataset (GSE82152 and E-MTAB-5151) including normal peripheral blood samples were used as the control group for differential expression analysis. We did not use these published data in the initial study because of the small sample size.

### Differential Gene Expression Analysis

First of all, non-peripheral-blood specific expression genes (no expression was detected in both control group and experimental group) were filtered. The limma package was used to find differential expression genes (DEGs) after normalization between two batches of data (Ritchie et al., 2015). Limma algorithm was originally developed for the analysis of microarray data, and its protocol for RNA-seq analysis also normalized the data using voom algorithm to process it into data similar to microarray for analysis (Ritchie et al., 2015). Thus, we used the GTEx dataset as the control group for a larger sample size. And the data of GTEx were normalized by voom algorithm and the batch effect of data were eliminated by the function named normalizeBetweenArrays. The standard of DEGs was an absolute log2 fold change greater than 2 and false discovery rate (FDR) *P*-value < 0.05.

### Functional Enrichment Analysis and Construction of Protein-Protein Interaction Network

To further explore the function of the DEGs above, the functional enrichment analysis was performed using the clusterProfiler including gene ontology (GO) term and the Kyoto Encyclopedia of Genes and Genomes (KEGG) pathway (Yu et al., 2012). String database was used to construct a protein-protein interaction (PPI) network based on the DEGs and the names of all the interacting proteins and the protein-coding genes were extracted from the network (Szklarczyk et al., 2019). Besides, non-parametric tests were used to assess the association between all DEGs and NeP.

### Processing of Single-Cell RNA-Seq Data

Raw reads in the sra file were first separated into pair-ended reads fastq files, which were trimmed to remove polyA tail sequence and the template switch oligo (TSO) sequence. Then, the clean reads were aligned to the hg38 human transcriptome (UCSC) and quantified by the Cell Ranger Single Cell Software Suite 3.3.1^4^.

For all 3,368 normal sequenced single PBMCs, cells with either fewer than 100,000 transcripts or fewer than 1,500 genes were filtered out. Besides, only the genes expressed in at least three single cells and their expression levels greater than 1 were considered for downstream analysis. Additionally, the Seurat method was applied to downstream analysis (Butler et al., 2018).

First, “vst” selection method was used to find variable genes, which were the input features for initial principal component analysis (PCA) (Butler et al., 2018). Then, the jackstraw analysis was performed to select the principal components (PCs) with *P*-values < 0.05 (Chung and Storey, 2015). Significant PCs were incorporated into further t-distributed Stochastic Neighbor Embedding (t-SNE) to identify different cell clusters with DEGs (resolution = 0.50). The standard of DEGs was an absolute log2 fold change greater than 0.50 and FDR value <0.05. Only the genes with an absolute log fold change greater than 0.5 and FDR *P*-value < 0.05 were selected as DEGs. The distribution and expression of top 10 DEGs were displayed by feature plots and heat maps, respectively. Additionally, scMatch (Hou et al., 2019), singleR (Aran et al., 2019), and CellMarker (Zhang et al., 2019) database were used as references for defining each cluster.

### Identification of the Significant Cellular Communication Among PBMCs

CellPhoneDB (Vento-Tormo et al., 2018; Efremova et al., 2019), a repository of ligand-receptor complexes and a statistical tool to predict the cell-type specificity of cell-cell communication via these molecular interactions, was performed to identify the ligand-receptor interactions and their cell localization among single PBMCs. The names of all the interacting proteins and the protein-coding genes were extracted from the network. Then, the Venn plot was used to illustrate the intersection of CellPhoneDB results and the PPI network.

### Validation by CIBESORT Algorithm

Cell type identification by estimating relative subsets of RNA transcripts (CIBERSORT) algorithm was used to characterize the cell composition from the complex tissues according to their gene expression profiles (Newman et al., 2015). Then the fraction of 22 types of immune cell was estimated in peripheral blood based on the normalized gene expression profiles. Samples with a CIBERSORT output of *P* < 0.05 were considered to be eligible for further analysis. Then, the Wilcoxon rank-sum test was applied to identify the immune cells with the fractions between peripheral blood samples from SCI patients and normal control samples. It should be pointed out that the CIBERSORT algorithm could correct for differences between different platforms and batches of data in the initial version (Newman et al., 2015). Additionally, the next generation CIBERSORT algorithm (CIBERSORTx) (Newman et al., 2019), including enable batch correction and disable quantile normalization algorithm, were applied to confirm the reliability of the results.

### Identification of the Potential Upstream Transcription Factors of Key Cellular Communication Genes

The DAVID database UCSC TFBS function module was used for TF enrichment analysis to identify target TFs among the DEGs. Differentially expressed TFs with enrichment analysis FDR < 0.05 were defined as significantly enriched TFs.

### GSVA and Co-expression Analysis

Gene Set Variation Analysis (GSVA) pathway analysis was performed to evaluate the expression levels of 185 KEGG pathways. The limma method was also used to find differentially expressed pathways between peripheral blood samples from SCI patients and normal control samples (Ritchie et al., 2015). The FDR *P*-value < 0.05, the log (fold-change) >0.5 or <−0.5 was defined a downregulated or upregulated pathway, respectively.

Eventually, a co-expression Pearson correlation analysis among significantly enriched TFs, key cellular communication genes and differentially expressed KEGG pathways.

### Reverse Transcription Quantitative Real-Time PCR (RT-qPCR) Assays and External Dataset Validation

Total RNA was isolated from was extracted from human whole blood of 16 patients with fractures complicated with SCI, 16 patients with fractures but no SCI and 8 normal adults, using QIAamp RNA Blood Mini Kits (Qiagen, catalog number 52304) according to the modified protocol of manufacturers. All cDNA generated from reverse transcription [PrimeScript RT Reagent Kit (Perfect Real Time) (Takara Bio)] was used for quantitative PCR analysis by ABI PRISM 7900 Sequence Detection System (Applied Biosystems, Foster City, CA, United States) and SYBR Premix Ex Taq (Tli RNaseH Plus) PCR Kit (Takara Bio). The relative expression levels of eight key genes (YY1, CEBPB, HAVCR2, LGALS9, MTOR, RPS6, RPS6KB1, RPS6KB2) and reference gene were determined by the 2^–ΔΔCt^ method. Kruskal–Wallis test was used to identity the statistical difference of gene expression among groups.

Additionally, since the platform effect and batch effect could inevitably affect the results of the differential genes, two Affy Primeview dataset (GSE82152 and E-MTAB-5151) including normal peripheral blood samples were used as the control group for differential expression analysis. The limma package was also used to find differential expression genes (DEGs) after normalization between two batches of data (Ritchie et al., 2015). The standard of DEGs was an absolute log fold change greater than 1 and FDR *P*-value < 0.05.

### Statistics Analysis

Two-sided *P*-value < 0.05 was thought to be statistical significance. All statistical analysis was conducted with R version 3.6.1 software (Institute for Statistics and Mathematics, Vienna, Austria)^5^ (Package: limma, Seurat, ggplot2, SingleR, reticulate, clusterProfiler, GSEABase, GSVA).

## Results

### Differential Gene Expression Analysis

**FIGURE 1:**
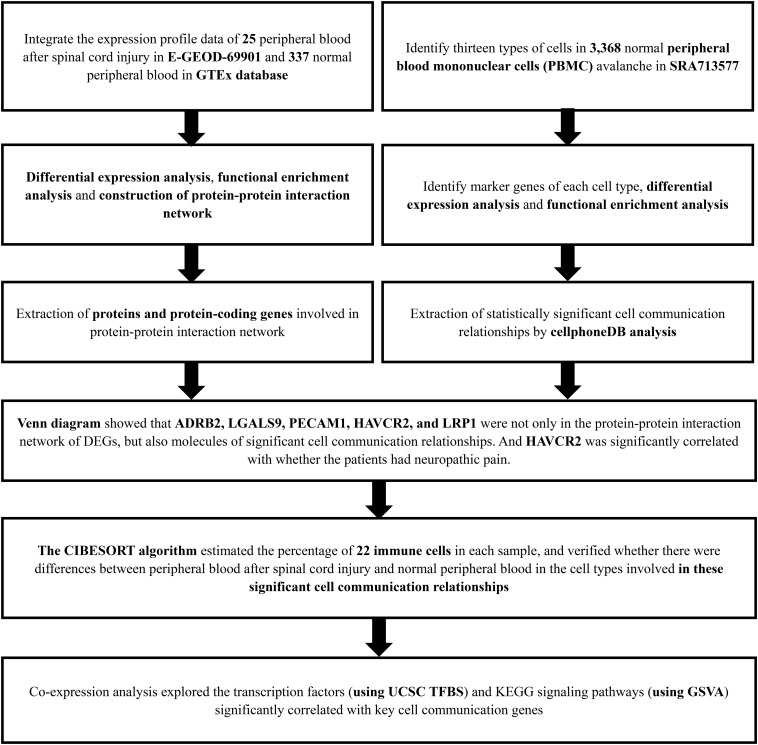
The flow chart of the analysis process. GTEx, Genotype-Tissue Expression; PBMC, peripheral blood mononuclear cell; SRA, Sequence Read Archive; CIBERSORT, Cell type identification by estimating relative subsets of RNA transcripts; KEGG, Kyoto Encyclopedia of Genes and Genomes; GSVA, Gene Set Variation Analysis.

**FIGURE 2:**
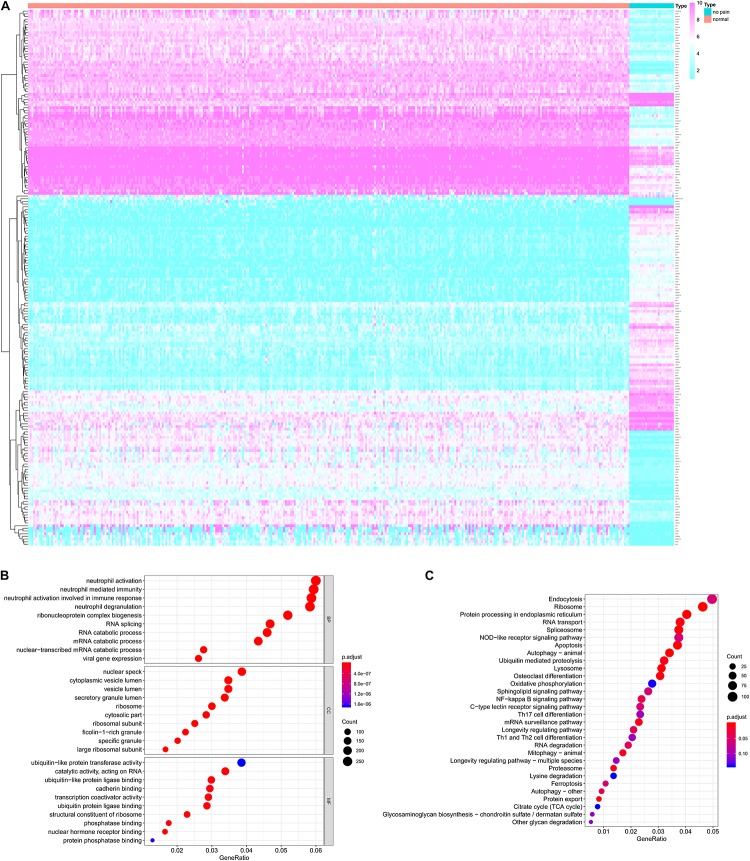
The differentially expressed genes (DEGs) between peripheral blood samples from spinal cord injury (SCI) patients and normal control samples **(A)** and the functional enrichment analysis for these DEGs in gene ontology (GO) terms **(B)** and Kyoto Encyclopedia of Genes and Genomes (KEGG) pathways **(C)**. **(A)** The heatmap of DEGs between peripheral blood samples from SCI patients and normal control samples. **(B)** The bubble plot of top 10 significant GO terms in biological process (BP), cellular component (CC) and molecular function (MF). **(C)** The bubble plot of top 20 significant KEGG pathways. DEG, differentially expressed gene; SCI, spinal cord injury; GO, Gene Ontology; KEGG, Kyoto Encyclopedia of Genes and Genomes; BP, biological process; CC, cellular component; MF, molecular function.

The analysis process of this study was presented in Figure 1. For identifying the significantly DEGs, we set the log (fold-change) >2.0 or <−2.0 and FDR < 0.05 as the cutoff and a total of 2,314 genes were identified as DEGs, including 1,152 upregulated ones and 1,162 downregulated ones (Figure 2A).

### Functional Enrichment Analysis and Construction of Protein-Protein Interaction Network

The functional enrichment analysis for these DEGs in GO terms and KEGG pathways were shown in Figures 2B,C, respectively. The biological process of GO terms analysis revealed the enrichment of some remarkable immune cell related terms. Additionally, “secretory granule lumen” “ubiquitin-like protein transferase activity” and “cadherin binding” were also significantly enriched as cellular component or molecular function, which might mean the active aberrant cellular communication in the peripheral blood of SCI patients (Figure 2B). The KEGG enrichment analysis also suggested some critical pathways were significantly associated with cellular communication, such as “Endocytosis,” “Protein processing in endoplasmic reticulum,” “RNA transport,” and “NF-κB signaling pathway” (Figure 2C).

String database was used to construct a PPI network based on the whole 2,314 DEGs, which included 4,807 PPI relationships related to 799 proteins (Supplementary Figure S1).

### The Gene Expression Landscapes of 3,368 PBMCs

**FIGURE 3:**
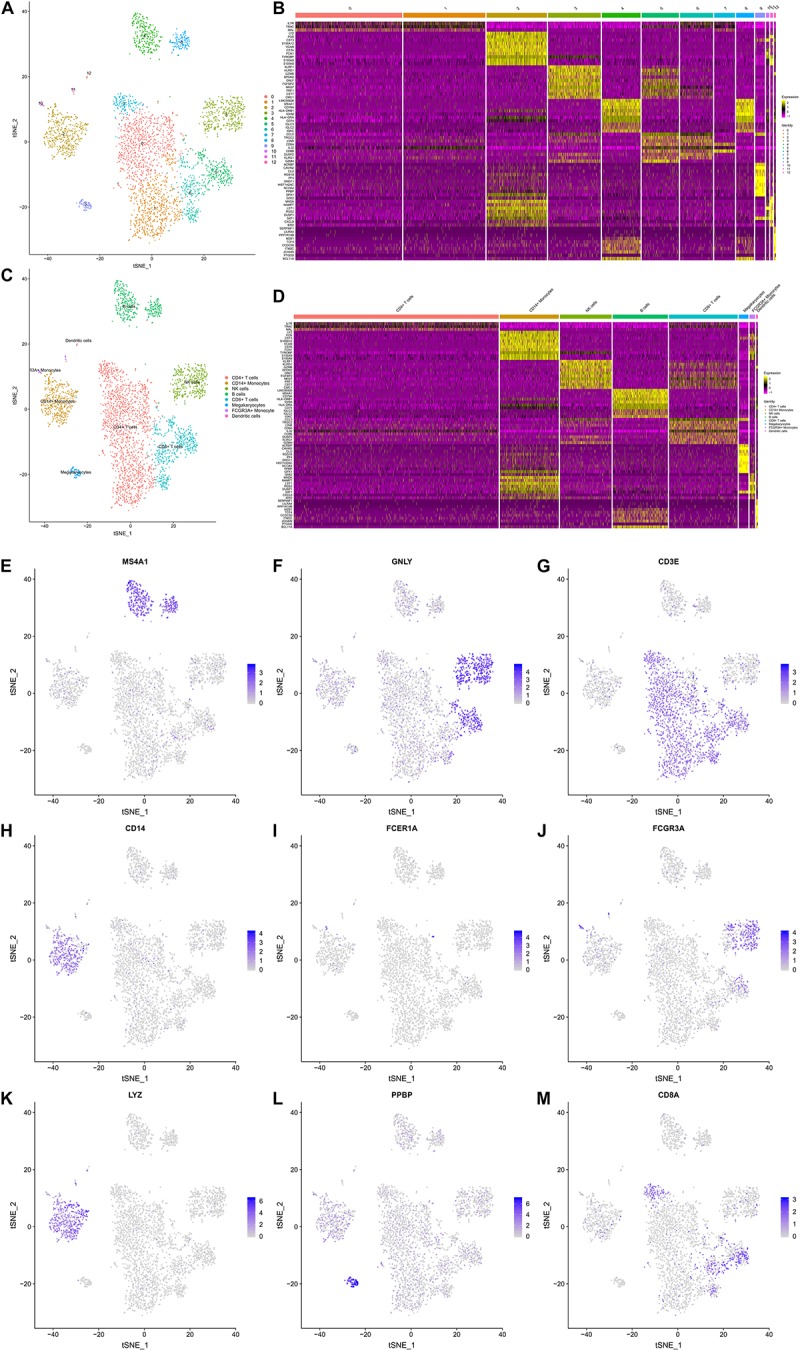
The Gene Expression Landscapes of 3,368 peripheral blood mononuclear cells (PBMCs). A t-distributed stochastic neighbor embedding (t-SNE) analysis was performed, which clearly identified 13 clusters **(A)** and 8 cell types (CD4+ T cells, CD14+ Monocytes, NK cells, B cells, CD8+ T cells, Megakaryocytes, FCGR3A + Monocytes, Dendritic cells) **(C)**. The expression levels the top 10 differentially expressed genes (DEGs) of each cluster **(B)** and cell type **(D)** are displayed in the heatmaps. **(E–M)** illustrate the feature plots of each cell type markers reported in the CellMarker database. PBMC, peripheral blood mononuclear cell; t-SNE, t-distributed stochastic neighbor embedding; DEG, differentially expressed gene.

A t-SNE analysis was performed and clearly identified 13 clusters and 8 cell types (CD4 + T cells, CD14 + Monocytes, Natural killer (NK) cells, B cells, CD8 + T cells, Megakaryocytes, FCGR3A + Monocytes, Dendritic cells) (Figures 3A,C). The expression levels of the top 10 DEGs in each cluster and cell type were displayed in Figures 3B,D, respectively. The feature plots of each cell type markers reported in the CellMarker database were presented in Figures 3E–M.

### Identification of Significant Cellular Communication Among PBMCs

**FIGURE 4:**
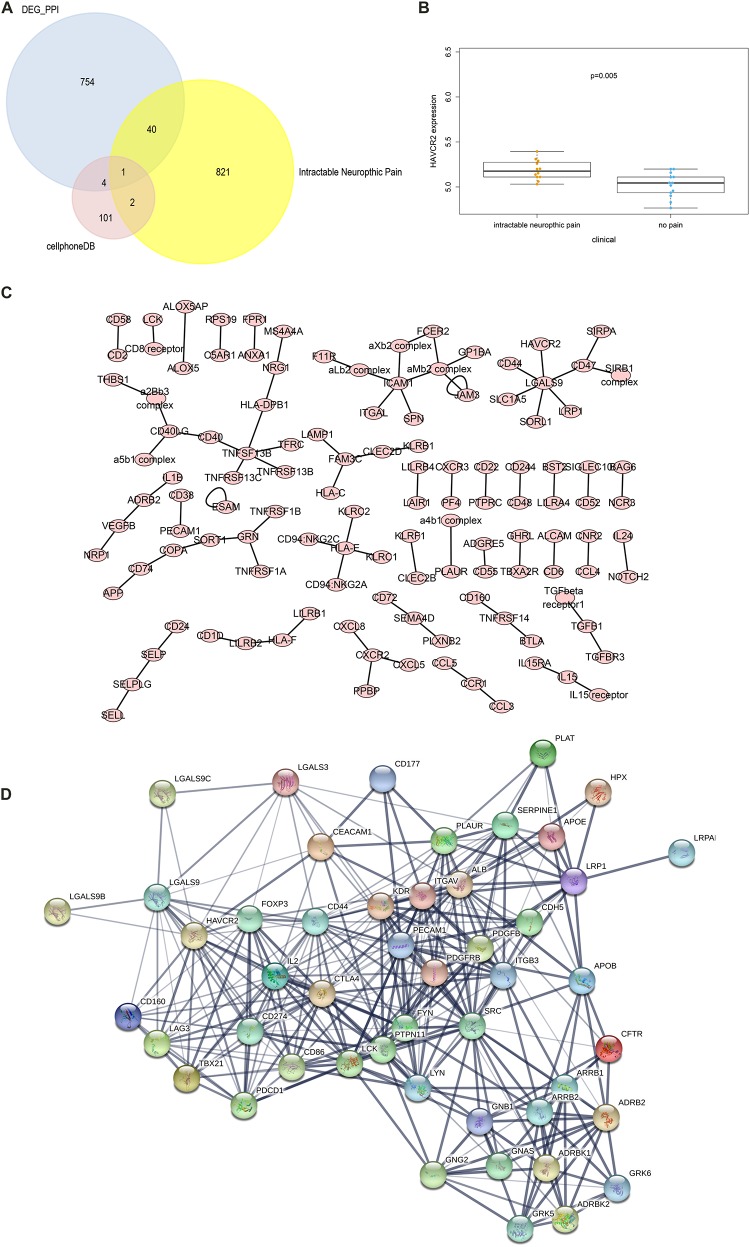
The results of the CellPhoneDB analysis and the Venn plot illustrating five proteins (ADRB2, LGALS9, PECAM1, HAVCR2, LRP1) that not only participated in significant ligand–receptor interactions in peripheral blood mononuclear cells (PBMCs) but Protein-Protein Interaction (PPI) network based on the differentially expressed genes (DEGs). **(A)** The Venn plot illustrating five proteins (ADRB2, LGALS9, PECAM1, HAVCR2, LRP1) that not only participated in significant ligand–receptor interactions in PBMCs but PPI network based on the DEGs, and only HAVCR2 was significantly associated with neuropathic pain (*P* = 0.005) **(B)**. **(C)** The network of 87 significant ligand–receptor interactions (related to 108 proteins); **(D)** PPI network illustrating the interactions among the ADRB2, LGALS9, PECAM1, HAVCR2, LRP1. PBMC, peripheral blood mononuclear cell; DEG, differentially expressed gene; PPI, Protein-Protein Interaction.

The CellPhoneDB analysis was performed to identify the ligand-receptor interactions and their cell localization among single PBMCs. A total of 87 significant ligand-receptor interactions (related to 108 proteins) and their cell localization were identified. Furthermore, 5 proteins (ADRB2, LGALS9, PECAM1, HAVCR2, LRP1) were identified in the overlap of proteins in the significant ligand-receptor interactions of PBMCs and PPI network based on the DEGs. Only HAVCR2 (Hepatitis A Virus Cellular Receptor 2) was significantly associated with NeP (*P* = 0.005) (Figures 4A,B). Besides, a total of 87 ligand-receptor interaction relationships and a new PPI network illustrating the interaction among the five proteins were shown in Figures 4C,D, respectively. Additionally, the results of CellPhoneDB analysis including ADRB2, LGALS9, PECAM1, HAVCR2, and LRP1 were presented in Table 1.

**TABLE 1:** The results of CellPhoneDB analysis involved ADRB2, LGALS9, PECAM1, HAVCR2, and LRP1.

**ID**	**Interacting A**	**Interacting B**	**Secreted**	**Rank**	**Cell localization**
CPI-SS098425155	ADRB2	VEGFB	TRUE	0.016	NK cells| Dendritic cells
CPI-SS0C6448B94	IL1B	ADRB2	TRUE	0.031	CD14+ Monocytes| NK cells, Dendritic cells| NK cells, CD14 + Monocytes| NK cells, Dendritic cells| NK cells
CPI-SS0E23CEB91	LGALS9	HAVCR2	TRUE	0.062	B cells| NK cells, CD14+ Monocytes| NK cells, Dendritic cells| NK cells, FCGR3A+ Monocytes| NK cells
CPI-SS0E0DEA7D5	PECAM1	CD38	FALSE	0.062	CD14 + Monocytes| NK cells, Dendritic cells| NK cells, FCGR3A + Monocytes| NK cells, Megakaryocytes| NK cells
CPI-SS0419B80C4	LGALS9	LRP1	TRUE	0.062	B cells| CD14+ Monocytes, CD14+ Monocytes| CD14+ Monocytes, Dendritic cells| CD14+ Monocytes, FCGR3A+ Monocytes| CD14+ Monocytes
CPI-SS002DF6C31	LGALS9	SLC1A5	TRUE	0.062	B cells| Dendritic cells, CD14+ Monocytes| Dendritic cells, Dendritic cells| Dendritic cells, FCGR3A+ Monocytes| Dendritic cells
CPI-SS09C52F54E	LGALS9	SORL1	TRUE	0.375	B cells| CD14+ Monocytes, B cells| CD14+ Monocytes, B cells| CD4 T cells, B cells| CD8 T cells, B cells| dendritic cells, B cells| FCGR3A+ Monocytes, B cells| NK cells, CD14+ Monocytes| CD14+ Monocytes, CD14+ Monocytes| CD4 T cells, CD14+ Monocytes| CD8 T cells, CD14+ Monocytes| Dendritic cells, CD14+ Monocytes| FCGR3A+ Monocytes, CD14+ Monocytes| NK cells, Dendritic cells| CD14+ Monocytes, Dendritic cells| CD4 T cells, Dendritic cells| CD8 T cells, Dendritic cells| Dendritic cells, Dendritic cells| FCGR3A+ Monocytes, Dendritic cells| Megakaryocytes, Dendritic cells| NK cells, FCGR3A+ Monocytes| CD14+ Monocytes, FCGR3A+ Monocytes| CD4 T cells, FCGR3A+ Monocytes| CD8 T cells, FCGR3A+ Monocytes| Dendritic cells, FCGR3A+ Monocytes| FCGR3A+ Monocytes, FCGR3A+ Monocytes| Megakaryocytes, FCGR3A+ Monocytes| NK cells
CPI-SS0703338F5	LGALS9	CD44	TRUE	0.500	B cells| B cells, B cells| CD14+ Monocytes, B cells| CD4 T cells, B cells| CD8 T cells, B cells| Dendritic cells, B cells| FCGR3A+ Monocytes, B cells| Megakaryocytes, B cells| NK cells, CD14+ Monocytes| B cells, CD14+ Monocytes| CD14+ Monocytes, CD14+ Monocytes| CD4 T cells, CD14+ Monocytes| CD8 T cells, CD14+ Monocytes| Dendritic cells, CD14+ Monocytes| FCGR3A+ Monocytes, CD14+ Monocytes| Megakaryocytes, CD14+ Monocytes| NK cells, Dendritic cells| B cells, Dendritic cells| CD14+ Monocytes, Dendritic cells| CD4 T cells, Dendritic cells| CD8 T cells, Dendritic cells| Dendritic cells, Dendritic cells| FCGR3A+ Monocytes, Dendritic cells| Megakaryocytes, Dendritic cells| NK cells, FCGR3A+ Monocytes| B cells, FCGR3A+ Monocytes| CD14+ Monocytes, FCGR3A+ Monocytes| CD4 T cells, FCGR3A+ Monocytes| CD8 T cells, FCGR3A+ Monocytes| Dendritic cells, FCGR3A+ Monocytes| FCGR3A+ Monocytes, FCGR3A+ Monocytes| Megakaryocytes, FCGR3A+ Monocytes| NK cells
CPI-SS014958F32	LGALS9	CD47	TRUE	0.500	B cells| B cells, B cells| CD14+ Monocytes, B cells| CD4 T cells, B cells| CD8 T cells, B cells| Dendritic cells, B cells| FCGR3A+ Monocytes, B cells| Megakaryocytes, B cells| NK cells, CD14+ Monocytes| B cells, CD14+ Monocytes| CD14+ Monocytes, CD14+ Monocytes| CD4 T cells, CD14+ Monocytes| CD8 T cells, CD14+ Monocytes| Dendritic cells, CD14+ Monocytes| FCGR3A+ Monocytes, CD14+ Monocytes| Megakaryocytes, CD14+ Monocytes| NK cells, Dendritic cells| B cells, Dendritic cells| CD14+ Monocytes, Dendritic cells| CD4 T cells, Dendritic cells| CD8 T cells, Dendritic cells| Dendritic cells, Dendritic cells| FCGR3A+ Monocytes, Dendritic cells| Megakaryocytes, Dendritic cells| NK cells, FCGR3A+ Monocytes| B cells, FCGR3A+ Monocytes| CD14+ Monocytes, FCGR3A+ Monocytes| CD4 T cells, FCGR3A+ Monocytes| CD8 T cells, FCGR3A+ Monocytes| Dendritic cells, FCGR3A+ Monocytes| FCGR3A+ Monocytes, FCGR3A+ Monocytes| Megakaryocytes, FCGR3A+ Monocytes| NK cells

### Validation by CIBESORT Algorithm

**FIGURE 5:**
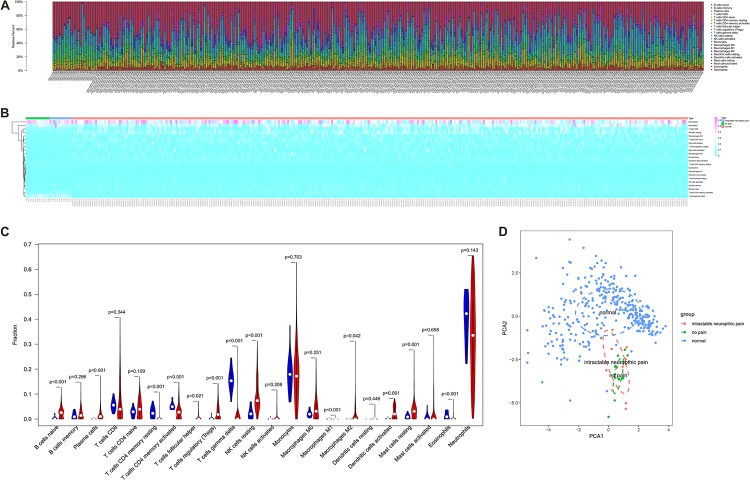
The composition **(A)** and heat map **(B)** of immune cells estimated by Cell type identification by estimating relative subsets of RNA transcripts (CIBERSORT) algorithm in peripheral blood samples from spinal cord injury (SCI) patients and normal control samples. **(C)** The violin plot identifying immune cells different from the two groups (the blue and red bar stand for SCI group and primary normal control samples, respectively). **(D)** The Principal Component Analysis (PCA) result of all samples suggesting the significant differences between the control group and the experimental group. CIBERSORT, cell type identification by estimating relative subsets of RNA transcripts; SCI, spinal cord injury; PCA, principal component analysis.

The fraction of immune cells in each sample estimated by CIBERSORT algorithm were displayed in Figures 5A,B. The results of the Wilcoxon rank-sum test suggested that the fractions of B cells naive (*P* < 0.001), plasma cells (*P* < 0.001), T cells CD4 memory resting (*P* < 0.001), T cells CD4 memory activated (*P* < 0.001), T cells follicular helper (*P* = 0.021), T cells regulatory (Tregs) (*P* < 0.001), T cells gamma delta (*P* < 0.001), NK cells resting (*P* < 0.001), macrophages M1 (*P* < 0.001), macrophages M2 (*P* = 0.042), dendritic cells activated (*P* = 0.001), mast cells resting (*P* = 0.001) and eosinophils (*P* < 0.001) had significantly different cellular fractions between peripheral blood samples from SCI patients and normal control samples (Figure 5C). These differential immune cells covered all the cell localizations of ADRB2, LGALS9, PECAM1, HAVCR2, and LRP1. In Addition, the PCA results of all samples suggested the significant differences between the control group and experimental group (Figure 5D). Besides, enable batch correction and disable quantile normalization algorithm of CIBERSORTx were used to eliminate platform effect and batch effect between different dataset. Wilcoxon rank-sum test suggested that the fractions of B cells naive (*P* < 0.001), plasma cells (*P* < 0.001), T cells CD4 memory resting (*P* < 0.001), T cells CD4 memory activated (*P* < 0.001), T cells follicular helper (*P* = 0.004), T cells regulatory (Tregs) (*P* < 0.001), T cells gamma delta (*P* < 0.001), NK cells resting (*P* < 0.001), macrophages M1 (*P* < 0.001), macrophages M2 (*P* = 0.002), dendritic cells activated (*P* < 0.001), mast cells resting (*P* = 0.001), and eosinophils (*P* < 0.001) also had significantly different cellular fractions between peripheral blood samples from SCI patients and normal control samples (Supplementary Figure S2).

### GSVA

**FIGURE 6:**
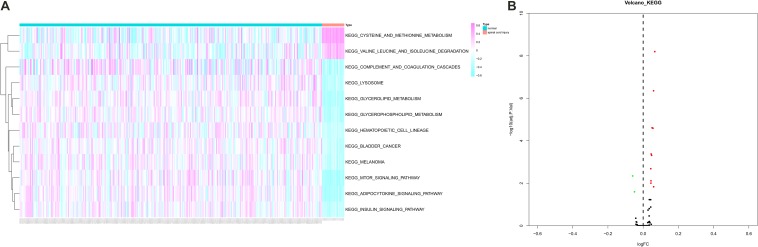
The heat map **(A)** and volcano plot **(B)** showing Kyoto Encyclopedia of Genes and Genomes (KEGG) 12 pathways were identified as differentially expressed pathways [Quantitative by Gene Set Variation Analysis (GSVA)] between peripheral blood samples from spinal cord injury (SCI) patients and normal control samples. KEGG, Kyoto Encyclopedia of Genes and Genomes; GSVA, Gene Set Variation Analysis; SCI, spinal cord injury.

Gene Set Variation Analysis was performed to estimate the expression levels of 185 KEGG pathways and 12 pathways were identified as differentially expressed pathways between peripheral blood samples from SCI patients and normal control samples (Figures 6A,B). Especially, some critical pathways were significantly associated with cellular communication and immune response such as “mTOR signaling pathway,” “complement and coagulation cascades pathway,” and “cysteine and methionine metabolism pathway.”

### Transcription Factor Enrichment Analysis

The TFs enrichment analysis was firstly performed based on all 2,314 DEGs and a total of 41 TFs were identified with FDR value < 0.05. Moreover, the enrichment analysis revealed that 10 significantly differentially expressed TFs might regulate the promoter regions of ADRB2, LGALS9, PECAM1, HAVCR2, and LRP1 (Table 2).

**TABLE 2:** Transcription factors enrichment analysis of ADRB2, LGALS9, PECAM1, HAVCR2, and LRP1.

**TF**	**Counts in DEGs**	**Percent of DEGs (%)**	**Fold Enrichment**	***P*-value**	**FDR**
ELK1	1150	49.69749352	1.211034109	7.59E-20	9.32E-17
SP1	588	25.41054451	1.288547397	9.6E-13	1.18E-09
RREB1	859	37.1218669	1.195598062	2.38E-11	2.93E-08
MZF1	1022	44.16594641	1.11604408	0.00000103	0.001270396
YY1	1468	63.43993086	1.076131756	0.00000131	0.001610992
CEBPB	1165	50.34572169	1.100197888	0.00000153	0.00187979
AHR	715	30.8988764	1.146008606	0.00000482	0.005924797
ARNT	1000	43.21521175	1.106636605	0.00000847	0.010404583
SRF	1466	63.35350043	1.068036681	0.0000121	0.014861228
GATA1	1675	72.38547969	1.051350602	0.0000355	0.043593096

*TF, transcription factor; DEG, differential expression genes; FDR, false discovery rate.*

### Co-expression Analysis

**FIGURE 7:**
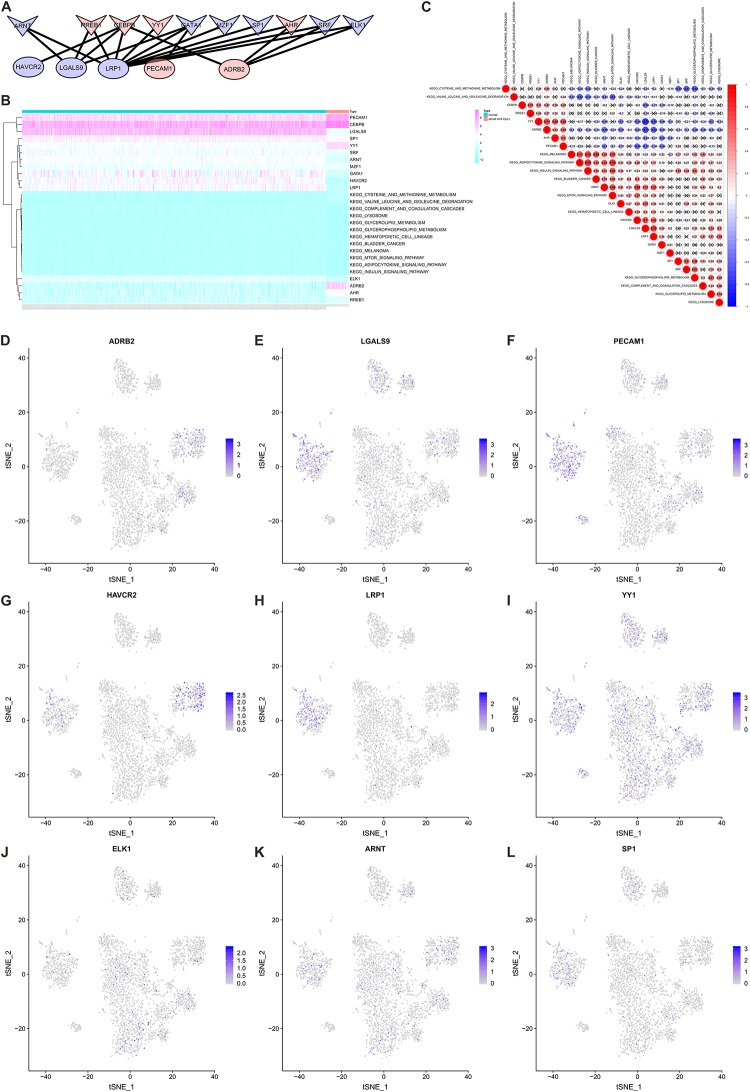
Construct regulation network and identify co-expression patterns among transcription factors (TFs), key cellular communication genes and differentially expressed Kyoto Encyclopedia of Genes and Genomes (KEGG) pathways. **(A)** The regulation network of TFs and key cellular communication genes (the V symbols represented TFs, the ellipses represented target DEGs; Red represented significant upregulated and blue represented downregulated). **(B)** The bi-clustering heatmap illustrating the expression levels of TFs, key cellular communication genes and differentially expressed KEGG pathways. **(C)** The co-expression heatmap illustrating the co-expression patterns of TFs, key cellular communication genes and differentially expressed KEGG pathways (in the co-expression heatmap, the transcription factor YY1 had significantly co-expression pattern with cellular communication receptor HAVCR2 (*R* = –0.54, *P* < 0.001), while HAVCR2 was also co-expressed with mTOR signaling pathway (*R* = 0.57, *P* < 0.001). **(D–L)** The feature plots showing the cellular localizations of the key TFs and target DEGs with co-expression patterns. TF, transcription factors; KEGG, Kyoto Encyclopedia of Genes and Genomes; DEG, differentially expressed gene.

**FIGURE 8:**
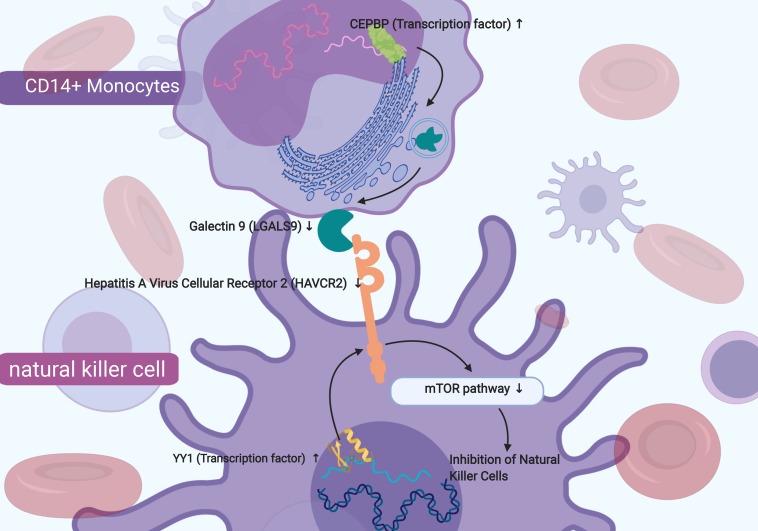
The sketch map of the signaling axis with the most significant co-expression pattern including YY1 (Yin and Yang 1 Transcription Factor), Hepatitis A Virus Cellular Receptor 2 (HAVCR2) and mTOR signaling pathway. In conclusion, this study inferred that the mechanism of YY1 regulating HAVCR2 and mTOR signaling pathway in the NK cells and the cellular communication between NK cells and CD14 + monocytes might play an important role in chronic phase of SCI and neuropathic pain.

A co-expression Pearson correlation analysis was used among significantly enriched TFs, key cellular communication genes and differentially expressed KEGG pathways. A regulation network was constructed based on TFs and key cellular communication genes (Figure 7A). The bi-clustering heatmap and co-expression heatmap illustrated the expression levels and co-expression patterns of the three components (Figures 7B,C). In the co-expression heatmap, the TF Yin and Yang 1 TF (YY1) had significantly co-expression pattern with cellular communication receptor HAVCR2 (*R* = −0.54, *P* < 0.001), while HAVCR2 was also co-expressed with mTOR signaling pathway (*R* = 0.57, *P* < 0.001). Besides, the TF CEBPB was significantly co-expressed with LGALS9 (*R* = −0.52, *P* < 0.001), which was the ligand of HAVCR2 and also co-expressed with HAVCR2 (*R* = 0.70, *P* < 0.001). Moreover, the cellular localizations of the key TFs and target DEGs with co-expression patterns showed that HAVCR2 and LGALS9 were located in NK cells and CD14 + monocytes, respectively (Figures 7D–L). Eventually, the sketch map of the signaling axis with the most significant co-expression pattern including YY1, HAVCR2, CEBPB, LGALS9, NK cell, CD14 + monocyte and mTOR signaling pathway was shown in Figure 8.

### Reverse Transcription Quantitative Real-Time PCR (RT-qPCR) Assays

**FIGURE 9:**
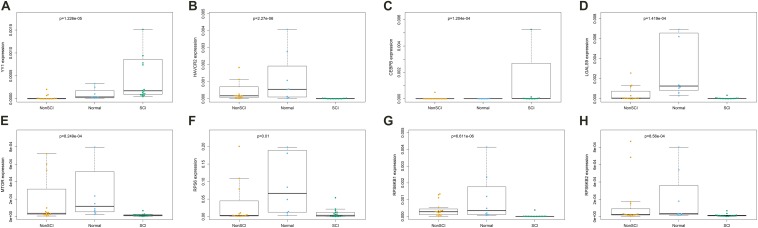
The results of Kruskal-Wallis test identifying the statistical difference of gene expression estimated by Reverse Transcription Quantitative Real-Time PCR (RT-qPCR) Assays. Total RNA was isolated from was extracted from human whole blood of 16 patients with fractures complicated with SCI, 16 patients with fractures but no SCI and 8 normal adults. The results of Kruskal–Wallis test suggested that transcription factor YY1 (**A**, *P* < 0.001) and CEBPB (**C**, *P* < 0.001) were upregulated in the peripheral blood of patients with SCI compared with patients with fractures but no SCI and normal adults. HAVCR2 (**B**, *P* < 0.001) and LGALS9 (**D**, *P* < 0.001) were also abnormally downregulated in peripheral blood of patients with SCI. Some key genes of the mTOR signaling pathway (MTOR, RPS6, RPS6KB1, RPS6KB2) were also identified to be significantly down-regulated in peripheral blood of patients with SCI (**E–H**).

Kruskal–Wallis test was used to identity the statistical difference of gene expression among groups. The results suggested that TF YY1 (Figure 9A, *P* < 0.001) and CEBPB (Figure 9C, *P* < 0.001) were upregulated in the peripheral blood of patients with SCI compared with patients with fractures but no SCI and normal adults. HAVCR2 (Figure 9B, *P* < 0.001) and LGALS9 (Figure 9D, *P* < 0.001) were also abnormally downregulated in peripheral blood of patients with SCI. Some key genes of the mTOR signaling pathway (MTOR, RPS6, RPS6KB1, RPS6KB2) were also identified to be significantly down-regulated in peripheral blood of patients with SCI (Figures 9E–H).

### External Dataset Validation

Additionally, since the platform effect and batch effect could inevitably affect the results of the differential genes, two Affy Primeview dataset (GSE82152 and E-MTAB-5151) including normal peripheral blood samples were used as the control group for differential expression analysis. Two volcano plots showing the results of differential expression analysis using two Affy Primeview dataset [GSE82152 (Supplementary Figure S4) and E-MTAB-5151 (Supplementary Figure S4)] including normal peripheral blood samples as the control group. And the differential expression analysis results of YY1, CEBPB, HAVCR2, LGALS9, MTOR, RPS6, RPS6KB1, and RPS6KB2 using GSE82152 and E-MTAB-5151 as control group were summarized in Supplementary Tables S1 and S2, respectively.

## Discussion

Spinal cord injury, one of the most devastating diseases, disrupts communication between the central and peripheral nervous, leading to the loss of essential neurological functions. Due to a large number of traffic and industrial accidents, the incidence rate of SCI is increasing rapidly around the world (Azarhomayoun et al., 2018; Musubire et al., 2019). Chronic phase of SCI may last a long period after the acute phase, which bring physically and psychologically devastating traumas to persons with SCI (Budh and Osteraker, 2007). SCI-related NeP is one of the most common symptoms in chronic phase and severely decreases the quality of life (Bryce et al., 2012). The molecular and cellular features often have some changes during the process of SCI-related NeP, and are often viewed as important predictors (Natinal Spinal Cord Injury Statistical Center [NSCISC], 2014). Thus, the DEGs and cellular communications in peripheral blood attract our interest, which is seldom reported by previous studies.

In the present study, an integrated transcriptome bioinformatics analysis based on bulk RNA sequence and single-cell RNA sequence was performed and the results inferred that the mechanism of YY1 regulating HAVCR2 and the downstream mTOR signaling pathway in the NK cells might be associated with SCI-related NeP. In addition, the cellular communication between NK cells and CD14+ monocytes might also play an important role in the SCI-related NeP. This hypothetical signaling axis might provide prognostic biomarkers and therapeutic targets for the SCI-related NeP.

YY1 was upregulated in the peripheral blood of patients with SCI in our study and almost distributed in all kinds of PBMCs. As a ubiquitously distributed TF, YY1 is involved in activating and repressing a diverse number of promoters. In addition, it modulates a variety of biological processes, particular in nerve and immune cells/tissues (Chen et al., 2017). NF-κB/YY1 signaling was reported to be associated with microglial activation in the progression of glaucoma, characterized with the progressive loss of retinal ganglion cells and optic nerve fibers (Lv et al., 2016). YY1 was expressed initially in pro-myelinating Sox9 + /Sox10 + Schwann cells (SCs) by E18.5 and continued to express in the early postnatal and adult SCs. Following acute nerve injury, YY1 expression was often maintained (Balakrishnan et al., 2016). In Stratton et al.’s (2017) study, YY1 was regarded as SC-associated proteins to promote axonal growth and regenerated axons and formed myelin following transplantation into the injured mouse sciatic nerve. Thus, we supposed that YY1 was a key TF in the SCI-related NeP.

HAVCR2 is also named T-Cell Immunoglobulin Mucin Family Member 3 (Tim-3). In this study, it was abnormally down-regulated in peripheral blood of patients with SCI and significantly correlated with the occurrence of SCI-related NeP. The protein coding by HAVCR2 belongs to the immunoglobulin superfamily and is involved in regulating innate and adaptive immune responses, usually mediating inhibition of target immune cells (Gorman and Colgan, 2014). HAVCR2 was reported to be abnormally expressed in T-cell lymphoma, acute myeloid leukemia, hepatitis A and injured nerve tissue. It regulated the activity of target immune cells through NF-κB signaling pathway, mTOR signaling pathway and RET signaling pathway (Prokhorov et al., 2015; Goncalves Silva et al., 2017; Avery et al., 2018). The role of HAVCR2 in nerve injury was shown in patients with spontaneous intracerebral hemorrhage whose increased HAVCR2 expression on CD14+ monocytes was associated with systemic inflammatory response and sub-acute brain injury (Xu et al., 2018). Besides our results, several previous studies revealed the close interaction between HAVCR2 and LGALS9. The interaction could inhibit the activity and promote the apoptosis of target cells, especially to the immune cells (Zhu et al., 2005; Clayton et al., 2014).

In our study, HAVCR2 was mainly distributed in NK cells in patients with SCI-related NeP, similar to the previous study which reported that HAVCR2 expressed on the surface NK cells was shown to act as a co-receptor to enhance IFN-gamma production in response to LGALS9 (Gleason et al., 2012). NK cells, originated from bone marrow derived lymphocytes, are crucial for immunoreaction against several infections and cancers (Nair et al., 2015). Post-SCI immunological changes impede neurological recovery and mediate common medical consequences of SCI, including NeP (Herman et al., 2018). It was also reported that NK cells were involved in peroneal nerve and their activation was essential in patients with traumatic SCI (Turker et al., 2012; Laginha et al., 2016; Xu et al., 2019). NK cells were observed a significant activation within 24 h after traumatic SCI regarding to the NK cell frequency and the presence of NK cells with the activated phenotype (Xu et al., 2019). During the post-acute and sub-acute phases after SCI, the function of NK cells was impaired (Laginha et al., 2016) and a marked downregulation of NK cell genes was found during chronic SCI (Herman et al., 2018). Therefore, we speculated that in peripheral blood of chronic SCI, HAVCR2 might act as a key receptor on the surface of NK cells and interact with ligand LGALS9 secreted by CD14+ monocytes, inhibiting NK cells through mTOR signaling pathway and ultimately predicting the occurrence of SCI-related NeP.

Furthermore, cell-to-cell communication across multiple cell types and tissues strictly governs proper functions of metazoans and extensively depends on the interactions between ligands and receptors (Hiramoto et al., 1993; Zhou et al., 2017; Cohen et al., 2018; Kumar, 2018; Mukherjee et al., 2018; Zhou et al., 2018). The specific communication utilized by the NK cell system and central nervous system results in conditioned response (Hiramoto et al., 1993). NK cells can engage the homotypic NK-to-NI cell interactions for optimal survival, activation and proliferation (Kim et al., 2014). However, the specific molecular mechanism utilized by the NK cell system and post-SCI central nervous system is not clearly understood. The PPI network is performed based on key genes associated with SCI-related NeP (YY1, HAVCR2, CEBPB, LGALS9), key members of mTOR signaling pathway (MTOR, AKT1, MAPK1, WNT4, PIK3CB) and the surface markers of NK cell (CD56, CD16, CD94, CD3, NKp46) (Supplementary Figure S3). Due to the extensive interaction between NK cell’s surface markers and mTOR signaling pathway, we hypothesized that mTOR signaling pathway might be associated with the NK cells in the SCI-related NeP.

mTOR plays a crucial role in many physiological functions of the CNS, including the regulation of neuronal cell growth and the development of axon and dendrite (Gong et al., 2015). Its function in SCI are associated with the time phase following SCI (Pouw et al., 2011). With regard to the acute phase of SCI, the mTOR signaling pathway participates in the regulation of neuronal apoptosis, autophagy, activation of macrophage/microglia, and local inflammatory response (Kanno et al., 2012). During the chronic phase, mTOR signaling pathway regulate the neuroregeneration and glial scar formation (Kanno et al., 2012). Thus, Rapamycin, an inhibitor of mTOR, is supposed to be a good treatment for SCI by preventing apoptosis of nerve cells (Yuan et al., 2016), promoting axonal regeneration and inhibiting the formation of glial scar (Kanno et al., 2012; Li et al., 2015). Moreover, mTOR regulates the development and maturation of T, B and NK cells and control the activation of macrophage/microglia (Powell et al., 2012; Xie et al., 2014; Almutairi et al., 2019; Rostamzadeh et al., 2019).

Moreover, in addition to HAVCR2 and LGALS9, the results suggested that ADRB2, PECAM1and LRP1 were also potential biomarkers associated with SCI. Adrenoceptor Beta 2 (ADRB2) in our study was also upregulated in the peripheral blood of patients after SCI and dominately distributed in the NK cells. ADRB2 (Adrenoceptor Beta 2) is a member of the G protein-coupled receptor superfamily. It is most abundantly expressed on the vasculature and modulated the release of nitric oxide and is involved in vascular function. Damage to the vasculature is universal consequences after SCI. Importantly, it has already been shown that ADRB2 agonists have neuroprotective effects and they improve the neurological and functional outcome, such as isoproterenol, salmeterol, and clenbuterol (Junker et al., 2002; Loy et al., 2002; Graumann et al., 2011). Treatment with the ADRB2 agonist can enhance the recovery in rats post-SCI (Zeman et al., 1999; Zeman et al., 2006; Brown et al., 2014). Scholpa et al. (2019) performed using an FDA-approved compound with the ability to be repurposed, reinforcing the potential clinical applicability of their findings and demonstrating the pharmacological activation of ADRB2 receptor for the treatment of SCI. However, it seemed no previous studies that reported the association between PECAM1/LRP1 and SCI. And due to the main findings of this study was that HAVCR2 might act as a key receptor on the surface of NK cells and interact with ligand LGALS9 secreted by CD14 + monocytes, inhibiting NK cells through mTOR signaling pathway and ultimately predicting the occurrence of SCI-related NeP. Thus, PECAM1/LRP1 were not discussed in details.

There are several unavoidable limitations in our study that should be taken into consideration. Firstly, although the results of the bioinformatics perdition suggested that the DNA binding domain (DDD) of YY1 could bind the promoter region of HAVCR2, no previous studies proved this interaction relationship by the direct mechanism assays. Secondly, the data released in public datasets are so limited that the clinicopathological features analyzed are not comprehensive, which might lead to potential statistical bias. Thirdly, due to the rapid progress of sequencing technology, there is heterogeneity between different batches and experimental platforms. Lastly, we must admit that there are two major limitation in this study, which are the bias between different platforms in the expression profile data and the absence of PBMC single-cell sequencing data of SCI patients. As the single-cell sequencing data originate from normal PBMCs, the results cannot well reflect the pathological changes of PBMCs following SCI. Therefore, a more comprehensive study is being conducting in our lab with data including bulk-RNA-seq and single-cell RNA-seq data of peripheral blood from patients with different time sequence SCI, and single-cell sequencing data of normal and injured spinal cord tissues in mice, which can not only validate the stability of the results in this study but also identify more biomarkers and therapeutic targets for SCI.

## Conclusion

In peripheral blood of chronic SCI, HAVCR2 might act as a key receptor on the surface of NK cells and interact with ligand LGALS9 secreted by CD14+ monocytes, inhibiting NK cells through mTOR signaling pathway and ultimately predicting the occurrence of SCI-related NeP. This hypothetical signaling axis may provide prognostic biomarkers and therapeutic targets for SCI-related NeP.

## Data Availability Statement

All datasets for this study are included in the ArrayExpress (E- GEOD-69901, https://www.ebi.ac.uk/arrayexpress/experiments/E-GEOD-69901/ and E-MTAB-5151, https://www.ebi.ac.uk/array express/experiments/E-MTAB-5151/), Gene Expression Omnibus (GSE82152, https://www.ncbi.nlm.nih.gov/geo/query/acc.cgi?acc=GSE82152), Genotype-Tissue Expression Portal (GTEx, https://commonfund.nih.gov/GTEx/) and Sequence Read Archive (SRA) (SRA713577, https://www.ncbi.nlm.nih.gov/sra/SRX4149408[accn]).

## Ethics Statement

The studies involving human participants were reviewed and approved by the Ethics Committee of Tongji Hospital, Tongji University School of Medicine. The patients/participants provided their written informed consent to participate in this study.

## Author Contributions

RH, TM, RZ, LZ, DS, HY, ZH, LC, and JZ: conception, design, data analysis, interpretation, manuscript writing, and final approval of the manuscript. RH, TM, and JZ: collection and/or assembly of data.

## Conflict of Interest

The authors declare that the research was conducted in the absence of any commercial or financial relationships that could be construed as a potential conflict of interest.

## Footnotes

1 https://www.ebi.ac.uk/arrayexpress/experiments/E-GEOD-69901/

2 https://commonfund.nih.gov/GTEx/

3 https://www.ncbi.nlm.nih.gov/sra/SRX4149408[accn]]

4 http://10xgenomics.com/

5 www.r-project.org

## Supplementary Material

FIGURE S1 The protein-protein interaction (PPI) network based on the all 2,314 differentially expressed genes (DEGs), which including 4,807 protein-protein interaction relationships related to 799 proteins. PPI, protein-protein interaction; DEG, differentially expressed gene.

FIGURE S2 The composition **(A)** and heat map **(B)** of immune cells estimated by the next generation Cell type identification by estimating relative subsets of RNA transcripts (CIBERSORTx) algorithm in peripheral blood samples from SCI patients and normal control samples; Enable batch correction and disable quantile normalization algorithm were used to eliminate platform effect and batch effect between different dataset. **(C)** The violin plot identifying immune cells different from the two groups (the blue and red bar stand for SCI group and primary normal control samples, respectively). **(D)** The Principal Component Analysis (PCA) result of all samples suggesting the significant differences between the control group and the experimental group. **(E)** The co-expression heatmap illustrating the co-expression patterns among 22 types of immune cells. CIBERSORT, cell type identification by estimating relative subsets of RNA transcripts; SCI, spinal cord injury; PCA, principal component analysis.

FIGURE S3 The protein-protein interaction (PPI) network comprised YY1, HAVCR2, CEBPB, LGALS9, key members of mTOR signaling pathway (MTOR, AKT1, MAPK1, WNT4, PIK3CB) and NK cell’s surface markers (CD56, CD16, CD94, CD3, NKp46). PPI, protein-protein interaction.

FIGURE S4 Two volcano plot showing the results of differential expression analysis using two Affy Primeview dataset [GSE82152 **(A)** and E-MTAB-5151 **(B)**] including normal peripheral blood samples as the control group. The standard of DEGs was an absolute log fold change greater than 1 and false discovery rate (FDR) *P* value < 0.05.

TABLE S1 The results of differential expression analysis of YY1, CEBPB, HAVCR2, LGALS9, MTOR, RPS6, RPS6KB1, and RPS6KB2 using GSE82152 and E-GEOD-69901 as control group and experimental group, respectively.

TABLE S2 The results of differential expression analysis of YY1, CEBPB, HAVCR2, LGALS9, MTOR, RPS6, RPS6KB1, and RPS6KB2 using GSE82152 and E-GEOD-69901 as control group and experimental group, respectively.

## Abbreviations

CIBERSORT: cell type identification by estimating relative subsets of RNA transcripts
DEG: differential expression gene
FDR: false discovery rate
GO: gene ontology
GSVA: Gene Set Variation Analysis
GTEx: Genotype-Tissue Expression Portal
KEGG: Kyoto Encyclopedia of Genes and Genomes
NeP: neuropathic pain
PBMC: peripheral blood mononuclear cells
PCA: principal component analysis
PPI: protein-protein interaction
RPKM: Reads Per Kilobase per Million
SCI: spinal cord injury
SRA: Sequence Read Archive
TF: transcription factor
Tim-3: T-Cell Immunoglobulin Mucin Family Member 3
t-SNE: t-distributed Stochastic Neighbor Embedding
TSO: template switch oligo.
