# Supplementary figures and images for: The Integrated Transcriptome Bioinformatics Analysis Identifies Key Genes and Cellular Components for Spinal Cord Injury-Related Neuropathic Pain
Source: Front Bioeng Biotechnol. 2020 Feb 19;8:101. doi: 10.3389/fbioe.2020.00101 (PMC7042182; doi:10.3389/fbioe.2020.00101)

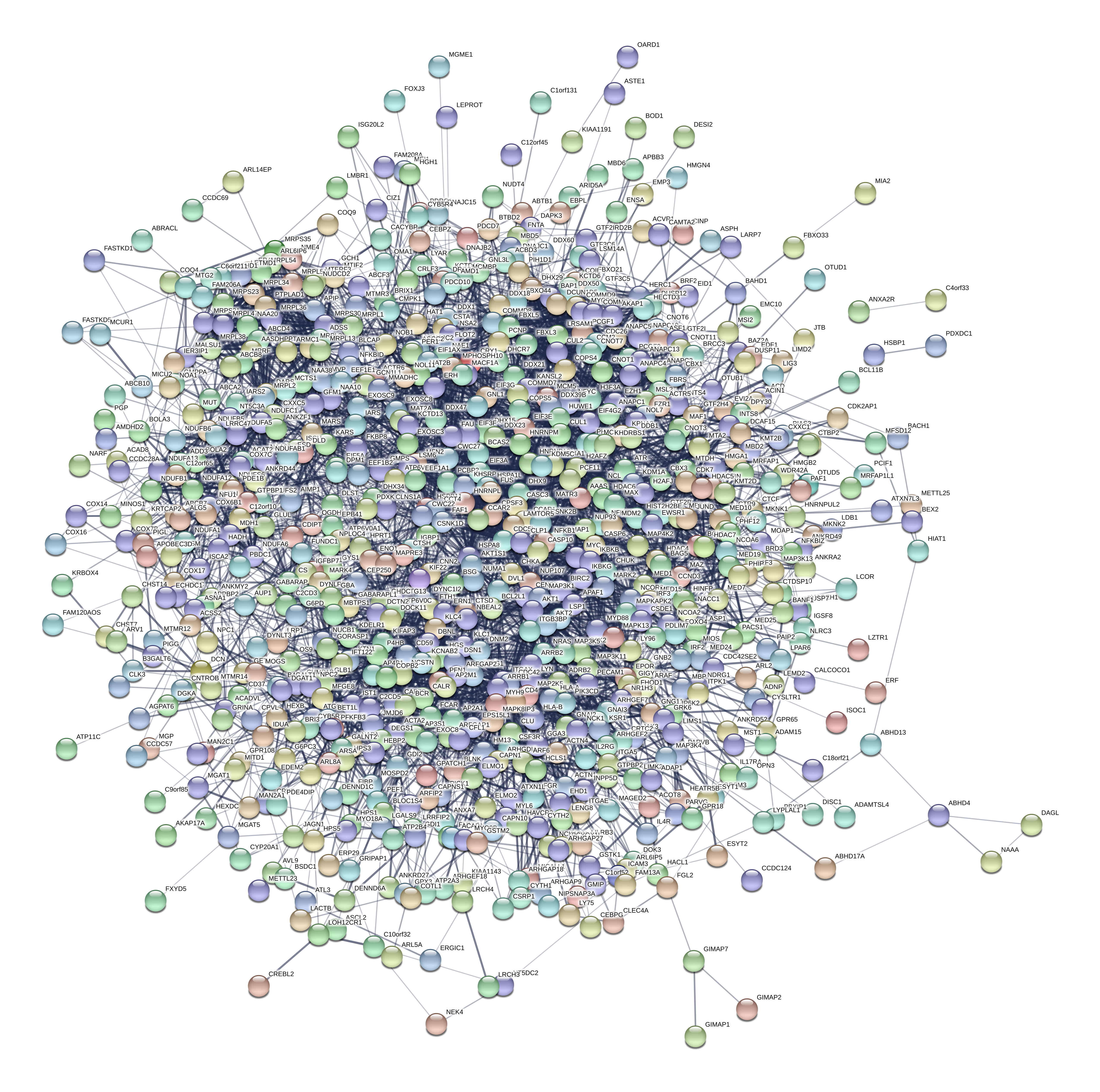

Supplement: FIGURE S1 — The protein-protein interaction (PPI) network based on the all 2,314 differentially expressed genes (DEGs), which including 4,807 protein-protein interaction relationships related to 799 proteins. PPI, protein-protein interaction; DEG, differentially expressed gene. [file Image_1.TIF]

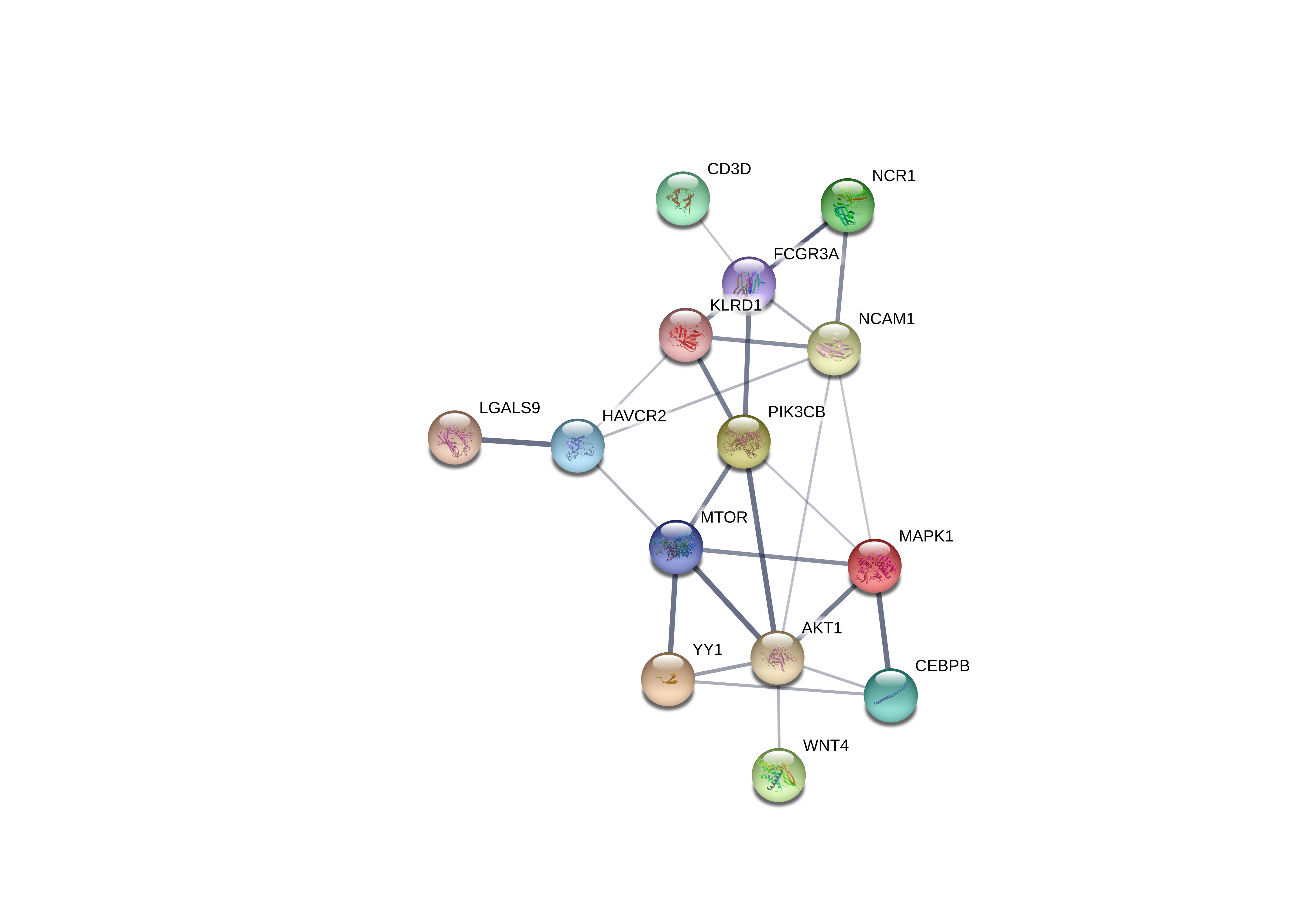

Supplement: FIGURE S3 — The protein-protein interaction (PPI) network comprised YY1, HAVCR2, CEBPB, LGALS9, key members of mTOR signaling pathway (MTOR, AKT1, MAPK1, WNT4, PIK3CB) and NK cell’s surface markers (CD56, CD16, CD94, CD3, NKp46). PPI, protein-protein interaction. [file Image_3.TIF]

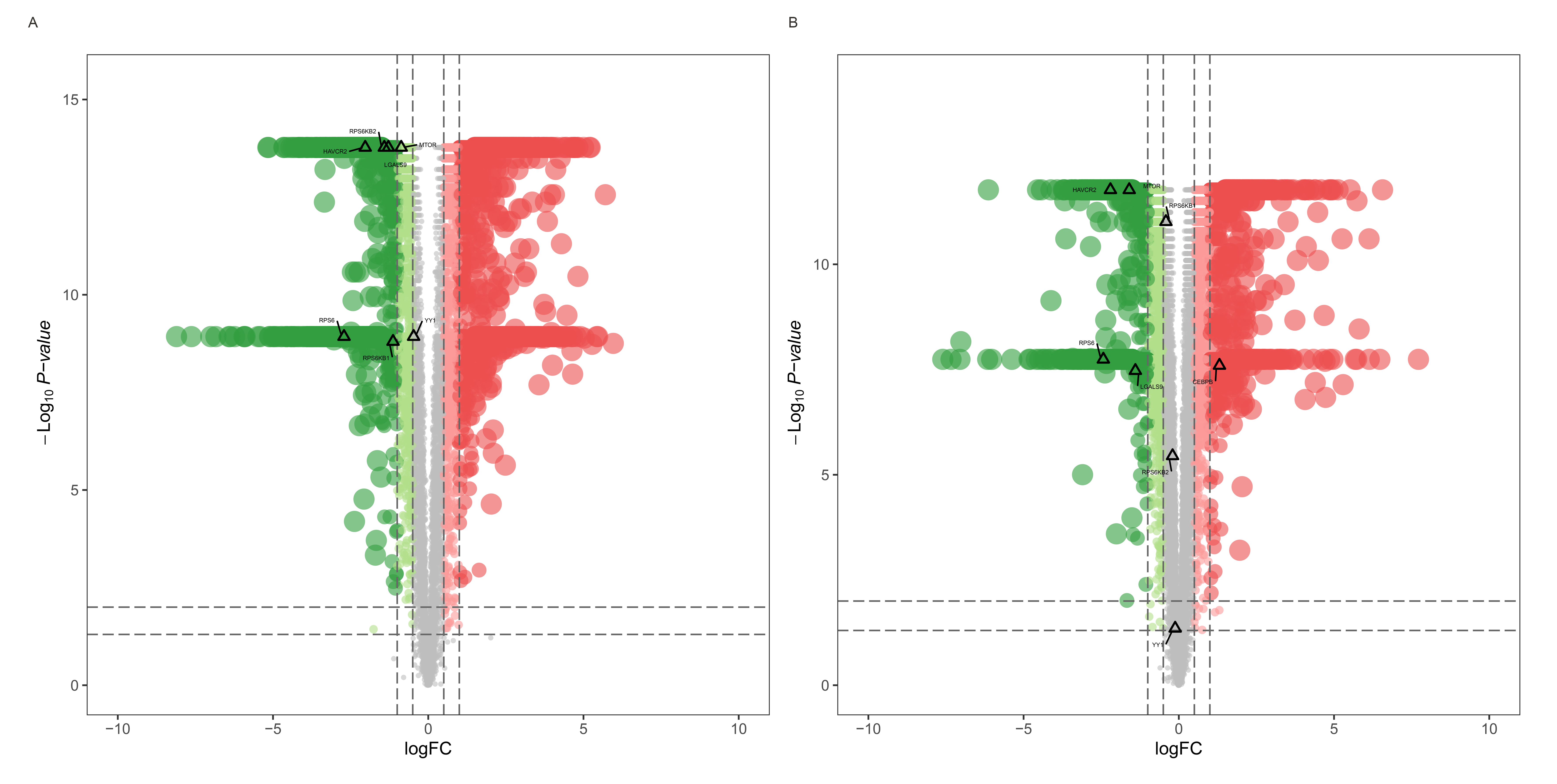

Supplement: FIGURE S4 — Two volcano plot showing the results of differential expression analysis using two Affy Primeview dataset [GSE82152 (A) and E-MTAB-5151 (B)] including normal peripheral blood samples as the control group. The standard of DEGs was an absolute log fold change greater than 1 and false discovery rate (FDR) P value < 0.05. [file Image_4.TIF]
