# Supplementary material for: The Integrated Transcriptome Bioinformatics Analysis Identifies Key Genes and Cellular Components for Spinal Cord Injury-Related Neuropathic Pain
Source: Front Bioeng Biotechnol. 2020 Feb 19;8:101. doi: 10.3389/fbioe.2020.00101 (PMC7042182; doi:10.3389/fbioe.2020.00101)
Supplement: TABLE S1 — The results of differential expression analysis of YY1, CEBPB, HAVCR2, LGALS9, MTOR, RPS6, RPS6KB1, and RPS6KB2 using GSE82152 and E-GEOD-69901 as control group and experimental group, respectively. [file Table_1.docx]

**Table S1** The results of differential expression analysis of YY1, CEBPB, HAVCR2, LGALS9, MTOR, RPS6, RPS6KB1 and RPS6KB2 using GSE82152 and E-GEOD-69901 as control group and experimental group, respectively.

| Gene symbol | Log2FC | P Value | FDR |
| --- | --- | --- | --- |
| CEBPB | 1.3087216 | 1.11E-08 | 2.53E-08 |
| MTOR | -1.597670084 | 2.88E-13 | 1.70E-12 |
| LGALS9 | -1.399169244 | 1.53E-08 | 3.36E-08 |
| RPS6 | -2.431075785 | 7.33E-09 | 1.81E-08 |
| RPS6KB1 | -0.415423707 | 2.02E-12 | 9.75E-12 |
| RPS6KB2 | -0.201316562 | 2.13E-06 | 3.56E-06 |
| YY1 | -0.117414924 | 0.038170509 | 0.044576766 |
| HAVCR2 | -2.202797086 | 2.88E-13 | 1.70E-12 |

**Abbreviations:** FC, Fold Change; FDR, False discovery rate.
