# Supplementary material for: The Integrated Transcriptome Bioinformatics Analysis Identifies Key Genes and Cellular Components for Spinal Cord Injury-Related Neuropathic Pain
Source: Front Bioeng Biotechnol. 2020 Feb 19;8:101. doi: 10.3389/fbioe.2020.00101 (PMC7042182; doi:10.3389/fbioe.2020.00101)
Supplement: TABLE S2 — The results of differential expression analysis of YY1, CEBPB, HAVCR2, LGALS9, MTOR, RPS6, RPS6KB1, and RPS6KB2 using GSE82152 and E-GEOD-69901 as control group and experimental group, respectively. [file Table_2.docx]

**Table S2** The results of differential expression analysis of YY1, CEBPB, HAVCR2, LGALS9, MTOR, RPS6, RPS6KB1 and RPS6KB2 using GSE82152 and E-GEOD-69901 as control group and experimental group, respectively.

| Gene symbol | Log2FC | P Value | FDR |
| --- | --- | --- | --- |
| CEBPB | NA | NA | NA |
| MTOR | -0.87055353 | 4.19E-15 | 1.69E-14 |
| LGALS9 | -1.282976721 | 4.19E-15 | 1.69E-14 |
| RPS6 | -2.712940585 | 6.54E-10 | 1.18E-09 |
| RPS6KB1 | -1.134618671 | 9.52E-10 | 1.58E-09 |
| RPS6KB2 | -1.415145785 | 4.19E-15 | 1.69E-14 |
| YY1 | -0.471929172 | 6.71E-10 | 1.18E-09 |
| HAVCR2 | -2.030543431 | 4.19E-15 | 1.69E-14 |

**Abbreviations:** FC, Fold Change; FDR, False discovery rate; NA, Not Available.
